# Editorial: Optimizing cardiovascular imaging for unusual clinical scenarios: a case-based approach

**DOI:** 10.3389/fcvm.2025.1615228

**Published:** 2025-05-14

**Authors:** Antonios Karanasos, Grigorios Korosoglou

**Affiliations:** ^1^ Department of Cardiology, Patras University Hospital, Patras, Greece; ^2^ Department of Cardiology, Vascular Medicine and Pneumology, GRN Hospital Weinheim, Weinheim, Germany; ^3^ Cardiac Imaging Center Weinheim, Hector Foundation, Weinheim, Germany

**Keywords:** echocardiography, cardiac magnetic resonance (CMR), cardiac computed tomography (CCT), coronary artery disease, tissue characterization, cardiac masses

**Editorial on the Research Topic**
Optimizing cardiovascular imaging for unusual clinical scenarios: a case-based approach

The landscape of cardiovascular medicine is continually advancing, yet the accurate diagnosis and effective management of complex cardiovascular diseases remain significant clinical challenges, requiring increasingly sophisticated diagnostic tools. Cardiovascular imaging serves as a cornerstone in this field, providing essential insights into cardiac anatomy, function, and tissue characterization. The evolution of technology during the recent decades offered several solutions that can assist the clinician in visualizing such complex clinical problems. Available cardiovascular imaging modalities range from echocardiography, which stands at the forefront as the first-line imaging modality, to newer modalities including cardiac magnetic resonance (CMR), computed tomography (CT), and nuclear techniques, each of which may offer its unique strengths. However, many of the modalities may have inherent limitations so that a multimodality cardiac imaging approach, leveraging the synergistic power of combining modalities, is often required in clinical practice. The current collection of articles showcases real-world examples where cardiovascular imaging modalities alone or in combination have contributed to the diagnosis of complex cardiovascular conditions by helping overcome diagnostic uncertainties, precisely characterizing pathology, and ultimately guiding optimal therapeutic strategies.

The prominent role of echocardiography in diagnosis of cardiovascular tumors was highlighted in the case of a diffuse myxoma on the mitral valve in a patient who presented with exertional dyspnea and chest discomfort (Dou et al.). Physical examination revealed a systolic murmur and echocardiography showed multiple masses with irregular shapes on the atrial sides of both the anterior and posterior mitral leaflets, which were then verified using transesophageal echocardiography. After surgical excision of the mitral valve, histology revealed the presence of a myxoma.

Additional information in patients with cardiac masses can be obtained by a multimodality approach employing CMR, a modality with prominent role in the diagnostic classification and risk stratification of patients with cardiac tumors (1, 2). This is also highlighted by the case report by Liu et al., where multi-modal cardiac imaging was employed for the evaluation of a suspected cardiac tumor. Echocardiography showed a hypoechogenic mass in the apical region of the left ventricle (LV), compatible with a cardiac tumor. CMR, however, indicated focal hypertrophy of the LV-apex, so that further specific treatment and surgical interventions could be deferred.

Similarly, multi-modality cardiac imaging using echocardiography, CMR and cardiac computed tomography angiography (CCTA) helped to identify the presence of a bronchogenic cyst, arising from the interatrial septum in a 42-year-old man who presented with symptoms of palpitation (Mingming et al.). The diagnosis of this rare congenital lesion was confirmed by histopathology after surgical resection of the tumor.

Previous studies demonstrated the potential of CMR for the early diagnosis cardiotoxicity due to different forms of chemotherapy (3). In this regard, an interesting case highlighted the role of CMR for the early diagnosis of immune checkpoint inhibitor (ICI)-myocarditis (Boussouar et al.). While ICIs have emerged as potent therapeutic agents in patients with cancer, they have been associated with immune-related adverse events also affecting the cardiovascular system, such as myocarditis. In this case, a patient with metastatic lung adenocarcinoma was treated using a combination regimen of monoclonal antibodies and ICI. Using CMR and mapping techniques and T2 values were both significantly prolonged, indicating myocardial edema. In addition, involvement of the diaphragm was noted, which contributed to the respiratory failure of the patient. After immunosuppressive therapy, CMR was used for monitoring response to treatment, demonstrating improvement in left ventricular ejection fraction and resolution of myocardial and diaphragmatic edema.

Apart from its well-defined role in the diagnosis of coronary artery disease, CCTA is gaining popularity as an adjunct to invasive coronary angiography for guiding treatment decisions and monitoring the impact of the treatment. In patients with prior coronary artery bypass grafting (CABG), a CCTA-directed strategy for performing coronary angiography may offer some advantages compared with the classic coronary angiography approach (4, 5). This is also highlighted in a case by Renker et al., where CCTA was used to monitor the result of a combined strategy for the treatment of an occluded venous graft, with administration of local thrombolysis following drug-eluting stent implantation to facilitate residual thrombus resolution. Follow-up images by CCTA demonstrated vessel patency without residual thrombus.

In another interesting case of a critically ill patient (Ostojic et al.), CCTA was used to diagnose a rare complication of pericardiocentesis. Specifically, a patient developed a large hemorrhagic effusion following pericardiocentesis, with CCTA disclosing contrast extravasation near the site of the puncture along with reduced perfusion of the anteropapillary muscle, thus suggesting a possible marginal branch perforation and dictating the need for an emergency cath-lab procedure for the management of the perforation, which was eventually confirmed by invasive coronary angiography. The perforation was managed successfully, sparing the patient from a more invasive procedure such as thoracotomy.

Moreover, CCTA has been utilized for performing computational fluid dynamics (CFD) in coronary arteries, thus providing mechanistic insights in the fields of atherosclerosis progression and vascular response to devices (6, 7). Similarly, CFD performed in models derived by CT angiography is being used to assess hemodynamics in patients treated for aortic dissection (8). The case by Iida et al. is a good example where CFD was performed to explain the mechanism of distal stent graft-induced new entry (dSINE) in a patient that had undergone frozen elephant trunk procedure for acute type A thoracic dissection. This patient was eventually treated by thoracic endovascular aortic repair (TEVAR). CT angiography performed prior to re-intervention showed the presence of true lumen stenosis with discrete elevations of wall shear stress, giving rise to the hypothesis that these hemodynamic disturbances may lead to aortic wall weakening, a potential risk factor for the progression or development of new dissections sites, as observed in this case.

The present case report collection highlights the role of current advances of non-invasive cardiac imaging for the diagnostic classification and risk stratification of patients with cardiac disorders. The implementation of these imaging modalities in the clinical routine and especially in challenging clinical cases, as these reported in our collection, can aid the timely and precise diagnosis of cardiac disorders, improving patient management and clinical outcomes.

## References

[1] Giusca S Mereles D Ochs A Buss S Andre F Seitz S Incremental value of cardiac magnetic resonance for the evaluation of cardiac tumors in adults: experience of a high volume tertiary cardiology centre. Int J Cardiovasc Imaging. (2017) 33(6):879–88. 10.1007/s10554-017-1065-7 28138817

[2] Giusca S Kelle S Korosoglou G. When tissue and outcomes are the issue. Cardiac magnetic resonance for patients with suspected cardiac tumours. Eur Heart J. (2021) 43(1):81–3. 10.1093/eurheartj/ehab625 34545406

[3] Giusca S Korosoglou G Montenbruck M Gersak B Schwarz AK Esch S Multiparametric early detection and prediction of cardiotoxicity using myocardial strain, T1 and T2 mapping, and biochemical markers: a longitudinal cardiac resonance imaging study during 2 years of follow-up. Circ Cardiovasc Imaging. (2021) 14(6):e012459. 10.1161/CIRCIMAGING.121.012459 34126756 PMC8208092

[4] Tsigkas G Toulgaridis F Apostolos A Kalogeropoulos A Karamasis GV Vasilagkos G CCTA-guided invasive coronary angiography in patients with CABG: a multicenter, randomized study. Circ Cardiovasc Interv. (2024) 17(9):e014045. 10.1161/CIRCINTERVENTIONS.124.014045 39286899

[5] Giusca S Schutz M Kronbach F Wolf D Nunninger P Korosoglou G. Coronary computer tomography angiography in 2021-acquisition protocols, tips and tricks and heading beyond the possible. Diagnostics (Basel). (2021) 11(6):1072. 10.3390/diagnostics11061072 34200866 PMC8230532

[6] Karanasos A Schuurbiers JC Garcia-Garcia HM Simsek C Onuma Y Serruys PW Association of wall shear stress with long-term vascular healing response following bioresorbable vascular scaffold implantation. Int J Cardiol. (2015) 191:279–83. 10.1016/j.ijcard.2015.04.231 25981369

[7] Hartman EMJ De Nisco G Kok AM Tomaniak M Nous FMA Korteland SA Wall shear stress-related plaque growth of lipid-rich plaques in human coronary arteries: an near-infrared spectroscopy and optical coherence tomography study. Cardiovasc Res. (2023) 119(4):1021–9. 10.1093/cvr/cvac178 36575921 PMC10153640

[8] Armour CH Menichini C Milinis K Gibbs RGJ Xu XY. Location of reentry tears affects false lumen thrombosis in aortic dissection following TEVAR. J Endovasc Ther. (2020) 27(3):396–404. 10.1177/1526602820917962 32364001 PMC7488817

